# Cancer survivors of gynecologic malignancies are at risk for decreased opportunity for fertility preservation

**DOI:** 10.1186/s40834-017-0039-4

**Published:** 2017-03-02

**Authors:** Wael H. Salem, Joe M. Letourneau, Jessica Chan, Sai-Wing Chan, Marcelle Cedars, Mitchell P. Rosen

**Affiliations:** University of California, San Francisco, Department of Obstetrics, Gynecology and Reproductive Sciences, San Francisco, CA USA

**Keywords:** Fertility preservation, Gynecologic cancer, Cancer survivorship, Counseling

## Abstract

**Background:**

Cancer survivors rate fertility as one of the most important determinants of their quality of life in the years after cancer treatment. We seek to describe the reproductive goals of women affected by gynecologic cancers and investigate their specific challenges during fertility preservation (FP) counseling.

**Methods:**

Univariate & multivariate logistic regression were used for quantitative analysis of objective FP counseling measures between women with gynecologic (GYN) and non-gynecologic (non-GYN) cancers from a cross sectional survey. Framework analysis was conducted on patient perception of physician-patient interactions.

**Results:**

Of the 2537 women contacted, 1892 responded and 1686 reported treatment with potential to impact fertility. Among women with GYN cancers 52% wanted future children. Women <35 years were interested in FP (74%). Women with Gyn cancers received less FP counseling than women with non Gyn cancer (OR 0.5 95% CI 0.4–0.6). Three hundred twenty-four patients gave qualitative answers. Patient identified barriers included incomplete FP information (59%), nondisclosure (29%), a disinterest in FP (5%), and a perceived urgency to start treatment (7%).

**Conclusions:**

Women with gynecologic cancers are less likely to be counseled about FP in comparison to women not affected by gynecologic cancers despite having similar fertility goals. We have identified patient perceived barriers to optimal FP counseling which may be improved upon to increase the value of FP and optimize quality of life for cancer survivors of gynecologic malignancies.

## Background

Comprehensive cancer care has made a transition to “quality survival” versus survival alone [1, 2]. Women affected by cancer in their reproductive years are in a particularly vulnerable position, having to balance their treatment plan and the possibility of diminished reproductive potential as cancer survivors [3]. The incidence of all cancers affecting women less than 50 years old has continued to rise. In 2011, women with gynecologic cancers accounted for 13% of the total incidence of women with cancers below the age of 50. Meanwhile, improvements in cancer screening and treatment modalities have led to increased cancer survival. Young women are living longer after their cancer treatment and thus the importance of quality of life during the years following is tantamount to the initial success of treatment itself [4, 5].

Cancer survivors rate fertility as one of the most important determinants of their quality of life in the years after treatment [6]. Among young cancer survivors who have never had children, 75% desire building a family [7]. Meanwhile, women who have lost their fertility as a result of their gynecologic cancers demonstrate depression, grief, stress and sexual dysfunction. While adoption or third party reproduction using donated gametes is an option, most patients express a desire to have their own biological children [8, 9]. It has been demonstrated that women may change their treatment decisions based on infertility concerns alone [10]. However, women affected by cancers are not consistently counseled about the risks of treatment to future fertility and/or referred to a FP expert [11–13].

Given the context and the conditions under which FP takes place, the patient, the providers and the medical system face numerous practical obstacles and psychological difficulties at the time of FP counseling. Previously-described barriers to FP include: suboptimal counseling, insufficient office time by the primary caregiver, a perception of patient disinterest, financial costs and insurance barriers, an urgency to initiate treatment, and a deficit in the primary provider’s knowledge base regarding FP [13, 14]. Interestingly, cost has not been shown to be the unique or overwhelming factor dissuading women from undergoing FP [15]. However, previous studies demonstrate important information deficits: more than 50% of physicians caring for women with cancer state that they rarely refer patients to a reproductive endocrinologist and only 38% of oncologists stated that they provided their patients with written information about FP [6, 16]. These layers of obstacles have led to FP utilization as low as 4% among reproductive aged women. Meanwhile, more recent studies demonstrate that among women receiving a FP referral approximately two-thirds opted for a FP treatment [17].

We set out to describe the reproductive health and FP counseling among women affected by gynecologic cancers. Our study seeks to describe the reproductive goals of women affected by gynecologic malignancies in addition to delineating the challenges confronted by women with gynecologic cancers in receiving optimal FP counseling. In uncovering their challenges, we offer specific counseling mechanisms in order to improve the quantity and quality of FP counseling and ultimately cancer survivors’ quality of life.

## Methods

We performed a retrospective survey study, using the California Cancer Registry (CCR) to sample women across the state of California. All study procedures were reviewed and approved by the IRB committee at the University of California, San Francisco Committee on Human Research.

### Study population

Women with gynecologic malignancies were contacted between December 2010 and February 2013. The detailed methods of the retrospective survey have previously been published and are available for reference [6, 18–20]. In brief summary, reproductive-age women from the California Cancer Registry (CCR) were sampled if they had a history of gynecologic cancer (cervical, ovarian, uterine/endometrial, vaginal/vulvar, and placental). This group was compared in this study to a control group within the CCR that consisted of reproductive age women (18-40 years) with leukemia, Hodgkin’s Disease, Non-Hodgkin Lymphoma, breast cancer and gastrointestinal cancers. These groups of cancers were chosen for this study because they are common gynecologic and non-gynecologic malignancies that may be treated with fertility-compromising surgery, chemotherapy, or radiation. Inclusion criteria included limitations on age (18 to 40 years of age at diagnosis) and time of diagnosis (1993 to 2007). The CCR updates its researchable database every three years, 2007 being the most recent at the time of initiation of this study. All available cases in the time period were selected for potential participation.

### Survey

A questionnaire was developed to query reproductive history before and after cancer treatment. The methodology regarding development and validity-assessment can be referenced in previously published work by our group [20]. The survey included information regarding FP counseling, patient described interest in future childbearing and background information regarding patient’s obstetric history. In the survey targeted at women with gynecologic cancers a separate free hand section was provided for the response to the question, “What did your doctor tell you about how cancer treatment could affect your ability to have children?”

### Data analysis

Survey data were merged with CCR data using a unique, anonymous identifier code. Statistically significant results were defined as *p* < 0.05 for a two sided *P* value. The Student’s *t*-test was utilized to compare responders versus non-responders. Multivariate regression analysis was conducted to determine the odds of FP counseling between groups. Statistical analyses were performed using STATA Version 11 (College Station, TX). Differences in diagnoses and demographic characteristics between women who did and did not respond were examined using CCR data.

A framework analysis was then carried out on all the qualitative responses to the free text response, “What did your doctor tell you about how cancer treatment could affect your ability to have children?” Two individuals reviewed the responses to categorize each response. A third individual was asked to review the response if non-concordant decisions were made by the first two. Patients were included in more than one category if they distinctly expressed barriers involving 2 or more categories. The methodology for a framework analysis was adhered to per established guidelines [21].

## Results

**Fig. 1:**
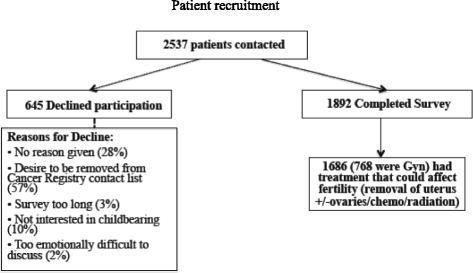
Patient recruitment

Among reproductive age women (18–40 years) in the CCR between 1993 and 2007, we contacted 2537 women and 1892 responded to the survey. The non-responders stated that the most common reasons for declining to complete the survey was the emotional difficulty, the length of the survey and a current disinterest in childbearing (Fig. 1). Among the 1892 responders, we identified 1686 (89%) as women who may have had their fertility impacted by their treatment and 768 of these had known gynecologic cancers. A total of 324 gynecologic cancer survivors who had treatment with potential to affect fertility articulated a written response to the question, “What did your doctor tell you about how cancer treatment could affect your ability to have children?”

Shown in Table 1 are the comparisons of the 1892 responders and 645 who declined participation, based on disease and demographic data in the CCR. There were small, but statistically significant differences between responders and non-responders in terms of age, time since diagnosis, age at treatment, socioeconomic status (calculated from median income and education for the census block group of residence at diagnosis) and stage of disease, as indicated by a SEER summary stage index (range of 0-in situ) to 7- metastatic). The age and childbearing desires of gynecologic and non-gynecologic cancer survivors who completed our survey are listed in Tables 2 and 3, respectively. Vulvar, vaginal, and placental cancer survivors were few in number (<15 total respondents each). Given their small sample sizes, these 3 cancer groups were excluded from further analysis. Ovarian cancer survivors tended to be the youngest, most likely to desire children, and most likely to conceive after treatment. Overall, few women attempted pregnancy (3–25%) despite a relatively high percentage of women who desired children after treatment (31–58%).

**Table 1:** Comparison of survey responders vs. declined

	Completed survey (*n* = 1892)	Declined (*n* = 645)	*p* ^†^
Age at diagnosis, years	32 · 4 (6 · 3)	34 · 4 (5 · 4)	<0 · 01
Age at survey, years	43 · 3 (7 · 9)	45 · 3 (6 · 9)	<0.01
Summary stage index^a^	3 · 3 (1 · 4)	2 · 8 (1 · 5)	<0 · 01
Socioeconomic Index^b^	2 · 5 (1 · 2)	3 · 0 (1 · 4)	<0 · 01
Time since diagnosis, years	10 · 8 (4 · 3)	10 · 7 (4 · 4)	0.63

†*p*-value from *t*-test comparing responders and non-responders

^a^Summary stage index = Surveillance Epidemiology and End Results staging index. Scores range from 0 (in situ) to 7 (metastatic)

^b^Socioeconomic index calculated from income, employment and education ranging from lowest to highest

**Table 2:** Characteristics of survey respondents that received treatment with potential to compromise fertility for GYN cancer

		Type of cancer				
	Total sample (*n* = 768)	Cervical (*n* = 218)	Ovarian (*n* = 244)	Uterine (*n* = 158)	Vulvar/Vaginal (*n* = 13)	Placental (*n* = 3)
Age at diagnosis, years, mean (SD)	33 · 4 (5 · 5)	33 · 3 (5)	31 · 7 (6 · 3)	36 · 1 (4 · 1)	37 · 9 (2 · 2)	33 (0)
Age at survey, years, mean (SD)	45 (6 · 9)	45 · 1 (6 · 5)	43 · 2 (7 · 7)	40 · 5 (7 · 1)	46 · 3 (4 · 4)	44 (0)
Years since diagnosis, mean (SD)	11 · 6 (4 · 2)	11 · 8 (4)	11 · 5 (4 · 3)	11 · 4 (4 · 5)	10 (3 · 1)	11 (0)
Children before treatment, %	65	73	55	64	77	100
Desiring children after treatment, %	52	49	58	51	31	0
Attempted pregnancy, %	13	10	25	3	17	0
Had children after treatment, %	8	7	15	1	8	0

**Table 3:** Characteristics of women reporting treatment with potential to impact fertility

		Type of cancer				
	Total sample (*n* = 918)	Leukemia (*n* = 121)	Hodgkin’s (*n* = 286)^a^	Non-Hodgkin’s (*n* = 169)^a^	Breast (*n* = 223)	Gastrointestinal (*n* = 108)
Age at diagnosis, years, mean (SD)	31.5 (6.7)	28.3 (7.2)	27.9 (6.2)	31.6 (6.0)	36.3 (4.0)	34.9 (4.6)
Age at survey, years, mean (SD)	40.9 (8.4)	37.0 (8.3)	36.5 (8.0)	40.5 (7.1)	47.1 (5.9)	44.6 (6.2)
Years since diagnosis, mean (SD)	9.6 (4.4)	8.7 (4.3)	8.6 (4.4)	8.9 (3.9)	10.8 (4.5)	9.7 (4.0)
Children before treatment, (%)	476 (52%)	46 (38%)	105 (37%)	88 (52%)	163 (73%)	76 (70%)
Desiring children after treatment, (%)	504 (54%)	71 (59%)	181 (63%)	82 (49%)	104 (47%)	61 (56%)

^a^Data missing from 11 patients with Hodgkin’s disease or non-Hodgkin lymphoma

Among those with gynecologic cancers and treatment with potential to affect fertility 52% explicitly stated an interest in future childbearing. Among women 18–35 years of age, 66% desired future children at the time of their diagnosis and 74% of those expressed an interest in FP. In the cohort of women older than 35, 35% still wanted future childbearing with 49% of those expressing an interest in FP. Nulliparous women of all ages were more likely to desire children (OR: 2.0 95% CI 1.4–2.7). These findings strongly support the presumption that women with gynecologic malignancies especially young women are interested in future childbearing and FP at the time of their diagnosis.

Among women desiring future childbearing, 51% of them were counseled regarding FP. The study group was then compared to a group of cancer survivors comprised from the California Cancer Registry who were affected by non-gynecologic cancers (Leukemia, Hodgkin’s, non-Hodgkin’s lymphoma, Breast or GI cancer). In comparison to women without gynecologic cancers, women with gynecologic cancers received significantly less counseling regarding FP (OR 0.5: 95%CI 0.4–0.6). Furthermore, this trend remained consistent among the groups of women most likely to desire future childbearing. Nulliparous women with gynecologic cancers were counseled at a lower rate than nulliparous women affected by non-gynecologic cancers (57% vs 50%: OR: 0.6 95% CI 0.4–0.86). They were also less likely to see a fertility specialist (4% vs 8% 95% CI 0.2–0.9). Women less than 35 years of age were less counseled if they had a gynecologic cancer than if they had another type of cancer (54% vs 67%: OR 0.5: 95% CI 0.4–0.7) and also less likely to see a fertility specialist (3% vs 8%, OR: 0.4 95% CI 0.2–0.8). Women with an explicit desire for future childbearing followed a similar pattern with regards to their FP counseling (51% vs 67%, OR: 0.5 95% CI 0.4–0.7). Rates of consultation with a reproductive specialist were similar in these groups (7%, OR: 0.98 95% CI 0.6–1.6). Given the low rates of FP counseling a further qualitative analysis was conducted to investigate possible explanations for the decreased counseling rates reported by cancer survivors of gynecologic cancers.

A content and framework analysis done on 324 women’s qualitative answers identified six major themes that emerged as significant barriers to adequate counseling. The majority of women (59%) noted physician truncation of information at the time of proposed treatment to be a barrier to further FP counseling. Namely, physicians were perceived to propose a treatment plan followed by a statement such as, “This will mean you won’t be able to have more children.” Patients expressed an understanding that the treatment will affect their fertility but were unaware of options for FP. Complete omission of information regarding fertility or FP was noted in 29% of poor FP counseling encounters. Incorrect knowledge based on the patient’s understanding was relatively less common (8%). A perceived urgency to start treatment was mentioned in 7% of responses, 6% of patients expressed a fear of introducing the conversation with their provider while a negative physician attitude or disinterest in FP accounted for 5%. Among all patients completing the qualitative response section 11% expressed a positive counseling experience.

Women who had gynecologic or non-gynecologic cancers identified the same primary sources for their FP counseling. Notably, the primary sources of information were the extended health care team (nursing, psychologist, etc.) followed by either the surgeon or medical oncologist. Women more likely to desire children and consider FP identified their partner (OR 2.2, 95% CI 1.03–4.7), family members (OR 2.2, 95% CI 1.0–5.2) or the internet (OR 4.8, 95% CI 1.4–16.8) as strong influences in their decision-making.

## Discussion

FP continues to be an underutilized asset in attaining the survivorship goals of reproductive aged women affected by cancer. It is well established that women who become infertile after cancer treatment experience regret and decreased quality of life [6, 10, 18]. Meanwhile, it has been demonstrated that women who merely undergo FP counseling have a decrease in regret and an increase in quality of life following their treatment [6]. Women place great importance on future childbearing even when confronted with the psychologically and physically jarring experience of a gynecologic malignancy. In our study population more than half of all the women with a gynecologic malignancy were interested in future childbearing. The inherent nature of a malignancy which directly affects a woman’s reproductive organs is also unique in its immediate effect on reproductive capacity and a woman’s perception of herself [22]. This constellation of short and long term ramifications necessitates a healthcare system in which optimal FP counseling is available for patients. In 2006, ASCO published guidelines strongly advocating for FP counseling for all patients of reproductive age [23, 24].

Despite various options for FP which offer an improved and expedited FP process, a host of barriers appear to remain in place leaving many women childless after cancer therapy [14, 25]. In this study, we set out to describe the reproductive goals of women affected by gynecologic malignancies and the specific counseling they receive in comparison to a control group of women with non-gynecologic malignancies. The last phase of the project evaluated the perceived barriers to receiving FP counseling per the patients. While some women affected by gynecologic cancers receive excellent FP counseling, the general trends evidenced by this research unfortunately demonstrate lower rates of FP counseling and decreased access to a fertility specialist in comparison with women not affected by a gynecologic cancer.

Our study demonstrates that women with gynecologic cancers have similar reproductive goals in comparison to women affected by other cancers. This is especially true of women younger than 35 years. Among women who represent those who would be most likely to desire future childbearing (age <35 or nulliparous) women were counseled at a significantly lower rate than those without gynecologic cancers. Furthermore, these women were less likely to consult with a fertility specialist. This study also sheds light on the group of women >35 years of age of which 49% stated that they would have been interested in FP at the time of their diagnosis.

These findings are somewhat at odds with the dialogue by the health care providers who care for women with gynecologic cancers [16]. Gynecologic oncologists stated that due to an Obstetrics and Gynecology training and an interdepartmental familiarity they were more likely to offer FP counseling [26]. Another recent study demonstrates that Gynecologic Oncologists are more confident in their knowledge of FP and thus they are more likely to discuss this with their patients [27].

Patients were very insightful as to the specific areas in which they found the counseling to be suboptimal. Most notably, patients felt that information was truncated leaving no room for further details or questions regarding FP. This may be explained by a low physician confidence discussing FP. A previous study demonstrated that only about half of surveyed oncologists had moderate or high confidence in their knowledge of FP options. Moreover, about 40% of oncologists do not feel that their responsibilities include FP counseling [27]. Complete omission of information was also common. Possibly as a result of these communication barriers regarding FP counseling, patients looked elsewhere for FP information. The extended healthcare team and the internet were the most common sources of additional FP information patients sited. In contrast, a previous study demonstrates that only 18% of oncologists gave their patients any resources regarding FP [27]. We suggest directing patients to preselected and reputable FP resource websites and to print out patient information sheets.

The evolution of FP and the increasing number of options for patients is accompanied with an increased complexity in counseling patients prior to treatment for gynecologic malignancies. ASCO has expanded the scope of providers it recommends to offer FP counseling. Specifically, the term “oncologists” was changed to “health care providers” from 2006 to 2013 [23, 24]. The findings in our study along with previous studies indicate that the scope of knowledge and time required for a complete discussion regarding FP counseling may be unrealistic within the context of addressing the primary oncologic needs. Our survey demonstrates a very low proportion of patients (11%) endorsing a positive counseling experience.

As this study’s participant population may have had cancer and treatment prior to the current availability of FP options, a time lag bias may have led to results which overstate the current lack of FP counseling. However, sustained low rates of FP referrals would argue that FP counseling remains suboptimal. Moreover, we would not anticipate a major change in the outcomes between our groups of interest based on a time lag bias. As with any retrospective survey study, the potential for recall bias is present. However, we do not anticipate that the recall bias would be significantly different between our groups of interest in this study. The use of the California Cancer Registry with a relatively high response rate allows this study to have a high degree of generalizability. This study also incorporates both a quantitative and qualitative element among the same group of patients to describe the specific differences among women affected by gynecologic cancers while also investigating the underlying reasons for variance between women affected by gynecologic cancers and those affected by other cancers.

Moreover, we identified specific barriers to counseling which may be resolved with improved health care team utilization, education and specific communication mechanisms. Physician truncation and omission of information are most likely due to insufficient clinic time and suboptimal physician confidence in their knowledge base [27, 28]. It would be beneficial to incorporate other members of the healthcare team who would be specifically instructed in basic FP counseling. Ongoing CME courses and intra institutional collaboration between oncology and reproductive endocrinology will further increase the dissemination of FP options. Communication regarding fertility impact and FP should end with an open ended question in order to decrease perceived truncation, omission and disinterest as these are the primary concerns of patients. Finally, an established system must ensure that patients undergoing cancer treatment receive an expedited referral and appointment with a reproductive endocrinologist should they choose to explore further options.

This study describes the fertility goals of survivors of gynecologic cancers and highlights findings which demonstrate suboptimal counseling among this population. We identify particular aspects of the physician-patient communication which may be improved from a communication and systems perspective. Additional avenues in which patients seek out information regarding FP are clearly highlighted based on our results. Utilizing improved communication mechanisms to open up the dialogue surrounding FP at first contact while increasing the counseling and referral rate to FP specialists will offer patients a spectrum of mental and reproductive health benefits in their life as cancer survivors.

## Conclusions

Women with gynecologic cancers are less likely to be counseled about FP in comparison to women not affected by gynecologic cancers despite having similar fertility goals. We have identified patient reported barriers which may be improved upon by increasing the utilization of other members of the healthcare team, increasing FP education and improving communication to increase the value of FP counseling at the time of cancer diagnosis. Ultimately, we must decrease barriers and improve FP counseling for women undergoing treatment of their gynecologic malignancy in order to fully address their reproductive goals and optimize their quality of life as cancer survivors.

## Abbreviations

CCR: California cancer registry
FP: Fertility preservation
GYN: Gynecologic
non-GYN: Non-gynecologic
